# From data to wisdom: A new era of scientific inquiry

**DOI:** 10.26508/lsa.202603865

**Published:** 2026-10-19

**Authors:** Nikolay V Dokholyan

**Affiliations:** Departments of Neurology, Neuroscience, Pharmacology, Biomedical Engineering, Immunology and Cancer Biology, The University of Virginia School of Medicine , Charlottesville, VA, USA

## Abstract

Rapid advances in high-throughput technologies and computational tools have ushered in a transformative era for scientific discovery. However, the acceleration of data generation has outpaced our conceptual frameworks for interpreting it. By examining current challenges in drug discovery, phenotypic screening, and model transferability, I argue that the future of scientific integrity depends on our ability to balance technical proficiency with the classical principles of hypothesis negation. Because the scientists who interpret data are themselves prone to bias and error, these safeguards must apply to human judgment as rigorously as to computational tools. I also confront a central tension: the mechanistic understanding I advocate may not scale to systems of cellular complexity. Finally, I propose a collaborative training approach that sustains both experimental excellence and theoretical depth. Each challenge maps onto the Data-Information-Knowledge-Wisdom (DIKW) hierarchy: failures at the D→I transition (underdetermined models), at I→K (undefined null hypotheses and black-box modeling), at K→W (non-transferable animal models and cross-domain blindness), or in the collapse of the entire chain when paradigm inertia forecloses the revision that wisdom demands.

## Abstract (precis)

This Commentary traces how failures along the data-information-knowledge-wisdom hierarchy mislead biomedical research and argues that rigor and falsification must govern human and machine judgment.

## Data Become Wisdom Only Through Deliberate Synthesis

In today’s scientific landscape, we are often flooded with data. Although data is fundamental to our work, we must recognize that, in its raw state, it does not constitute information. This progression from data (D) to information (I) to knowledge (K) to wisdom (W) echoes the familiar “DIKW” hierarchy (1, 2), a framing justly criticized for treating these transitions as automatic. I invoke it here not as a ladder climbed through accumulation, but as a sequence of distinct synthetic acts, each requiring an effort that the previous stage does not provide. Borrowing loosely from information theory, we can think of raw, unstructured data as containing much more than is relevant to any given question. Extracting information largely means reducing its dimensionality: retaining the variables that matter and discarding those that do not. This reduces uncertainty only if we retain and discard the right data, and judging which data to retain and discard requires assumptions that may themselves be wrong, a point I return to below. I use this as a metaphor rather than a formal claim: in Shannon’s sense (3), information and entropy are closely related. The intuition nonetheless holds: organizing data into information requires work, both time and methodological rigor.

As we move further up the hierarchy, information matures into *knowledge* when we begin to connect disparate observations into a coherent framework. Linnaeus’s *Systema Naturae* organized an enormous body of observations into a hierarchical classification of living things (4), turning data into information. Darwin’s theory of evolution by natural selection (5) explained why such a hierarchy should exist at all: nested groups are exactly what descent with modification produces. A catalog of species, however complete, predicts little; an explanation tells us what to expect next. In drug discovery, the same distinction separates a list of compounds that are active in an assay from an understanding of why they are active, and only the latter allows a medicinal chemist to rationally design the next molecule. Yet the ultimate goal of science is not merely knowledge, but wisdom — the ability to synthesize views across multiple domains to understand the nuanced phenomena of the real world. Wisdom is a slow process; it requires deliberately boiling knowledge over time. In our pursuit of rapid discovery, we must ensure that we do not bypass these critical stages of synthesis in favor of sheer volume.

## More Data Is Not More Information

An abundance of data is often mistaken for an abundance of information. Cellular imaging and omics experiments generate terabytes of data, but how we extract information from it shapes what we learn, and the extraction process can carry hidden biases from the scientist’s assumptions, models, and expectations. A high-throughput screen can test hundreds of thousands of compounds and return thousands of apparent “hits,” yet many of these are pan-assay interference compounds that appear active through aggregation, redox cycling, or reactivity with assay components rather than genuine engagement of the target (6). Data are abundant; information about which compounds truly bind is scarce, and it emerges only with appropriate controls and counter-screens. Most critically, one step is routinely skipped: checking whether the amount of data matches the model’s complexity and can yield unambiguous answers. In DIKW terms, this is a failure at the very first transition: without sufficient data to span the parameter space of the model, the transition never occurs — we remain at the level of data, however vast, rather than ascending to information.

Consider building a model of a drug’s toxicity. This requires understanding the drug’s impact across roughly 20,000 proteins and a comparable number of genes, plus regulatory elements, physiological states (such as liver enzyme levels and the microbiome), and systemic factors (such as neuronal wiring or an immune state) that influence side effects. Naively, the space of relevant molecular configurations is astronomically large: even a coarse enumeration over ∼2 × 10^4^ proteins, a comparable number of genes, ∼10^4^ regulatory elements, and a handful of physiological and microbiome states yields well beyond Ω ≈10^16^ combinations. It is tempting to conclude that one would need a commensurate number of measurements, and that terabytes are therefore self-evidently too few. I resist that inference, because it misdescribes how modeling actually works: no one characterizes such a system by tiling its Cartesian product with data points. Modern statistical learning routinely advances when the number of nominal parameters vastly exceeds the number of observations, thanks to regularization, hierarchical and Bayesian priors, sparsity, parameter sharing, and low-dimensional structure; over-parameterized models can fit and even generalize, even when a naive degrees-of-freedom count suggests they should not.

The real difficulty is therefore not arithmetic but epistemic: it concerns what the data can and cannot tell us. Recovering a system’s causal structure from observational data is generically an ill-posed inverse problem: many distinct parameterizations, and indeed many distinct mechanisms, can reproduce the same observations equally well (7). What breaks this degeneracy is never data volume alone; it is the structure one is willing to assume before fitting begins: the priors, constraints, and mechanistic commitments that make a high-dimensional system identifiable in the first place. Those assumptions, not the measurements, do the decisive work, and they are precisely the mechanistic content I contend is indispensable. The danger of the data deluge is therefore not a shortage of data points, but the illusion that the data speak for themselves, when in fact every well-posed answer depends on assumptions that are often unstated and unexamined. These assumptions are made by fallible people, and they are frequently wrong. Mistaking the consequences of one’s assumptions for the verdict of the data, and hiding that step behind a large N, is a widely neglected failure and a major source of irreproducible conclusions.

## Knowledge Is Only as Strong as Its Null Hypothesis

Synthesizing information into knowledge depends on the models used to interpret it, specifically, the null hypothesis that serves as a baseline reference, and the alternative models that could explain the same information. The null hypothesis determines how much knowledge can be gained: a weak one yields little. For instance, positing that a drug binds with extreme specificity to a single protein is easily refuted by many well-designed experiments, so disproving it tells us little beyond the obvious fact that the drug has side effects. By contrast, a null hypothesis that incorporates all plausible alternative explanations yields richer, more precise knowledge about how the drug acts. Too often, studies leave both the null and alternative hypotheses undefined, undermining their reliability. Within the DIKW paradigm, the I→K transition is precisely what a well-constructed null hypothesis operationalizes: it defines the conditions under which organized observations (information) can be forged into explanatory understanding (knowledge).

## Wisdom Lies in Recognizing Shared Principles Across Domains

Wisdom emerges when we discern the fundamental principles that lie beneath our separate domains of knowledge, when disparate fields, each with its own vocabulary and phenomena, turn out to be speaking variations of the same underlying truth. Consider that the prices of stock market options, as described by the Black-Scholes equation, obey the very same mathematical form as the position and velocity of a particle buffeted by drag and random forces, captured by the Fokker-Planck equation. A trader’s portfolio and a pollen grain drifting in water would seem to share nothing, yet both are governed by the same law. The reason is that both are expressions of a single deeper principle: the mathematics of stochastic processes. In DIKW terms, the K→W step is therefore not the accumulation of more knowledge within one domain but the recognition of shared structure across domains, a synthesis that no quantity of data, and no single discipline working alone, can supply. Wisdom in this sense is genuinely emergent: it cannot be reduced to any of the knowledge stocks that compose it, and its attainment requires the deliberate, collaborative juxtaposition of fields that would otherwise remain strangers to each other.

## Easy-to-Use Models Demand More Scrutiny, Not Less

The democratization of machine learning and artificial intelligence has provided us with powerful new lenses through which to view our data. However, the ease with which these tools can be applied can occasionally mask the complexity of the underlying mathematics. When the barrier to entry is low, models risk being applied as “black boxes,” without a full accounting of the assumptions built into their architecture. In drug discovery, for example, sophisticated docking algorithms can predict high-confidence binding interactions even when the target protein’s structural integrity has been intentionally compromised or “shredded” in simulation (8). This suggests that, in such cases, the models may respond to sequence-based correlations rather than the fundamental physics of molecular recognition. These instances of non-transferable knowledge remind us that our computational tools are only as robust as the biological, physical, and chemical context we provide. In DIKW terms, this is the I→K transition short-circuited: a black-box model processes inputs into outputs without ever constructing the explanatory causal structure that constitutes genuine knowledge. The result is a system that can reproduce information within its training distribution but cannot generate the understanding needed to reason outside it: pattern without principle, correlation without comprehension.

## Human Judgment Is Indispensable, and Fallible

The same critique applies to the scientists who build and interpret these models. If the ease with which computational tools can be applied masks the complexity of the underlying mathematics, the ease with which a human scientist can reach a simple conclusion equally masks the complexity of the underlying biology. The framework of assumptions needed to turn data into information is a human construction, and it can also be wrong. In this respect, scientists resemble the deep neural networks criticized above: trained on a limited and biased sample of experience, we tend to discount observations that do not fit our expectations and to amplify those that do. This tendency, known as confirmation bias (9), contributes to the poor reproducibility of many published findings (10). Entire fields have been held back for decades because influential figures were confidently wrong. Lipmann’s “squiggle” notation for the “high-energy phosphate bond” (11), popularized in biochemistry textbooks for generations, encouraged the misconception that energy is stored in a particular chemical bond, whereas the free energy released by ATP hydrolysis is a property of the entire reaction, including reactants, products, and solvent. Similarly, when Avery, MacLeod, and McCarty showed in 1944 that DNA is the transforming principle (12), many leading geneticists, including members of the influential phage group, remained unconvinced, in part because the prevailing tetranucleotide hypothesis portrayed DNA as a monotonous polymer too simple to carry genetic information. Nothing in the DIKW hierarchy guarantees that the people climbing it are wise.

I, therefore, do not claim that human scientists are wiser than their tools. Scientific research benefits when scientists understand how data are generated and how complex interpretive models can be. However, this understanding is useful only when scientists exercise sound judgment and question their own assumptions. The safeguards against human and machine error are largely the same: state assumptions explicitly, define null hypotheses and alternative models before analyzing the data, blind analyses where possible, replicate findings independently, and build teams diverse enough in training and outlook that one person’s blind spot is another’s obvious flaw. Human and machine fallibility are best treated not as rivals but as complementary sources of error, each capable of checking the other.

## A Corrected Marker Is Not a Cured System

The traditional approach to studying biological systems is to develop a mechanistic understanding of the molecular players involved in a particular phenomenon. This approach, while arduous and complex, preserves the causal logic of the processes underlying the phenomenon under study. While the mechanistic approach has yielded a wealth of knowledge, understanding an organism’s function on a grand scale remains elusive. This raises a genuine tension. On the one hand, phenotypic approaches risk mistaking correlation for causation, and mechanistic insight is indispensable; on the other hand, the cell may be so complex that a full mechanistic description is unattainable in practice. Such complexity may make the cell non-separable: its key state variables may not split cleanly into parts that can be measured and manipulated independently, as the classical reductionist program assumes. If so, the problem is not only that the cell is complicated, but that one variable cannot be changed without changing others, as I illustrate in the homeostatic example below. My resolution of the tension is thus not “more mechanism” versus “more data,” but a disciplined hybrid: phenotypic breadth to generate hypotheses, mechanistic depth to test them, and explicit acknowledgment of which coordinates remain unmeasured.

Phenotypic approaches have emerged as a convincing alternative to mechanistic ones, offering a more holistic view of cellular behavior. The premise of this approach is to view the system as a whole by utilizing large-scale omics experiments. In principle, if we could monitor *all* players in the system and collect sufficient data, we should be able to understand and predict cellular behavior and extrapolate that knowledge to the organismal level. The devil is in the details: the complexity of such screening is on the same scale as mentioned above, 10^16^. The massive amount of data required for holistic modeling is also error-prone, and errors accumulate in large numbers.

Currently, collecting this amount of data in a statistically meaningful way is not feasible, and, as argued above, even abundant sampling would not by itself resolve the underlying ill-posedness. Hence, scientists often rely on a set of measured markers. While the ability to restore a pathological phenotype to a healthy appearance is a significant achievement, it is vital to distinguish between a corrected marker and a cured system. A cellʼs phenotype is a high-dimensional function of its entire internal state, which is analogous to a wave function or a partition function in physics. When we monitor a specific set of variables, such as gene expression or protein phosphorylation, we observe only a small subset of coordinates on a vast manifold. It is theoretically possible to restore these visible coordinates while other, unmonitored parameters diverge further from the norm. In other words, we tend to work with known knowns, but ignore unknown unknowns, and even known unknowns.

Consider a simple example. Suppose a dataset shows a relationship between shoe size and lifespan. People with larger shoe sizes might appear to have shorter lifespans because both variables are associated with sex (men, on average, have shorter lifespans than women). This does not mean that changing someoneʼs shoe size would change how long they live. This example is only an illustration, not an empirical claim. Similarly, we should not mistake correlation for causation. However, correlations can still be useful. Causal inference methods, including the potential-outcomes framework (13), graphical causal models (14), and natural experiments such as Mendelian randomization, can help identify causal relationships in observational data. These methods require clear assumptions, carefully chosen controls, and much more work than identifying a simple correlation. They remain underused, partly because they are rarely taught or funded.

## Example: Networks Reconfigure Around Targeted Interventions

To formalize this phenomenon, consider a directed network in which an input signal *X*_*0*_ = 10 branches into two downstream coordinates: a monitored primary marker *X*_*1*_ and an unmonitored hidden coordinate *X*_*2*_. Both feed into a pathological output phenotype *Y*, whose state is a weighted sum of its inputs: *Y = w*_*1*_*_γ_X*_*1*_ *+ w*_*2*_*_γ_X*_*2*_, with weights *w*_*1*_*_γ_* = 8 and *w*_*2*_*_γ_* = 2. At the pathological baseline, both coordinates are elevated by the input, with *X*_*1*_ = 10 and *X*_*2*_ = 10. The resulting output is *Y* = (8 × 10) + (2 × 10) = 100, the full pathological value. Because *X*_*1*_ is the marker clinicians observe, a targeted intervention is designed to suppress it, say, by 75%, driving *X*_*1*_ down to 2.5. Holding everything else fixed, the output falls to *Y* = (8 × 2.5) + (2 × 10) = 40. The monitored marker now reads as corrected, and the system appears to be in remission.

The network, however, is plastic. Over time, the unmonitored coordinate *X*_*2*_ adapts homeostatically, rising from 10 to 40 to compensate for the loss elsewhere. With *X*_*1*_ still at its corrected value of 2.5 but *X*_*2*_ now diverged to 40, the output returns to *Y* = (8 × 2.5) + (2 × 40) = 100, exactly its pathological baseline. The marker we watch remains reassuringly low, yet the disease state has been fully restored through a channel we never measured. Inhibiting a single observed node corrects the appearance of disease without correcting the disease itself, because unmonitored coordinates reconfigure to conserve the overarching pathological state. Viewed through the DIKW lens, the clinician has information (X_1_ is suppressed) but not knowledge (the network has reconfigured around the intervention), and certainly not the wisdom to anticipate how the unmeasured manifold will respond over time. The example, thus, maps the entire DIKW chain onto a single clinical scenario: data without contextual interpretation is merely noise; information without causal structure is dangerously incomplete; knowledge without cross-domain breadth cannot anticipate systemic compensation; and wisdom, the recognition that every targeted intervention perturbs an adaptive whole, is precisely what this field must cultivate.

## Transferability Between Models Is a Hypothesis, Not a Given

The reliance on standardized animal models, such as the laboratory mouse, has been a foundation of biomedical research. These models provide a controlled environment that is essential for reproducibility. However, we must also consider the environmental plasticity of the systems we study, particularly the immune system. Consider a thought experiment involving identical twins: one raised in an ultra-hygienic, controlled environment, and the other in a diverse, high-exposure natural setting. Their immune systems, though genetically identical, would develop vastly different records of environmental adaptation. When we attempt to transfer findings from a rodent model raised in a sterile, incubated environment to a geographically and environmentally diverse human population, we must do so with extreme care. The transferability of these models is not a given; it is a hypothesis that requires its own rigorous validation. In DIKW terms, what transfers between experimental systems is not raw data, nor even information, but structured knowledge, and even then, the hierarchy warns us that knowledge derived in one biological context does not automatically constitute wisdom about another.

Behavioral and cognitive models also require caution. Past experiences can shape how an organism responds to each new event. Yet researchers rarely measure this history or ensure that it is similar across subjects. The scent of agarwood illustrates this point: no description can fully convey it to someone who has never experienced it. By contrast, someone who has smelled agarwood before will experience it differently from someone encountering it for the first time, partly because they recognize the scent. This issue is especially important in psychedelic research. A person with previous psychedelic experience may recognize the effects, creating a lasting source of bias. The practical issue, however, is not the philosophy of subjective experience; it is that two genetically identical organisms can differ in an unrecorded variable, their accumulated experience, and so respond differently to the same perturbation. A laboratory animal raised in a controlled, limited environment is not equivalent to a free-living animal with a wide range of experiences. We should therefore test whether behavioral findings apply across these subjects rather than assume that they do. In the DIKW framework, this is a claim about the transfer of knowledge into wisdom. This reliance on what can be known only through direct encounter, rather than through description, is what ties the problem to the foundations of the empirical method. Francis Baconʼs formulation of the scientific method emphasizes the importance of both intellectual models and experiential testing in understanding reality: “There are and can be only two ways of searching into and discovering truth. The one flies from the senses and particulars to the most general axioms… the other derives axioms from the senses and particulars, rising by a gradual and unbroken ascent, so that it arrives at the most general axioms last of all. This is the true way, but as yet untried” (15). Unfortunately, many models are presented as absolute truths about a phenomenon or experiment, when in fact they are merely simplified representations that can be misleading if taken too literally.

## Progress Requires Trying to Refute Our Favorite Ideas

Classical scientific inquiry is rooted in the principle of falsification. As proposed by Popper, the strength of a theory is measured not by how many times it has been validated, but by its ability to survive repeated, rigorous attempts at refutation: “Every genuine test of a theory is an attempt to falsify it, or to refute it. Testability is falsifiability” (16). In modern practice, however, the pressure to produce positive results can sometimes lead to a focus on validation at the expense of negation. The history of the amyloid cascade hypothesis in Alzheimerʼs research provides a thoughtful example of this dynamic (17, 18, 19, 20, 21). When clinical outcomes did not align with initial predictions, the hypothesis was not rejected but was instead refined, shifting focus from fibrils to oligomers to specific membrane-bound species. While such refinement is a natural part of the scientific process, we must remain vigilant against “paradigm inertia.” Such repeated, ad hoc rescue of a theory — what Popper called a “conventionalist stratagem” and what Lakatos later characterized as the mark of a degenerating research program (22) — is precisely the failure mode falsification is meant to guard against. In DIKW terms, paradigm inertia represents a failure to move from knowledge to wisdom. A field may continue building knowledge that supports its dominant framework while ignoring the framework’s limitations and suppressing alternatives. Wisdom requires openness to new explanations. Falsification is therefore essential because it allows existing ideas to be challenged and enables the transition from knowledge to wisdom.

Falsification is easier to support in principle than to practice. Few scientists actively try to disprove their own favored theories. This reluctance reflects both funding pressures and human nature. Kuhn offered a cautionary response to Popper. During periods of “normal science,” researchers work within an accepted framework. They often explain away conflicting evidence rather than treat it as a reason to reject the framework. According to Kuhn, a framework is usually abandoned only when conflicting evidence creates a crisis and a credible alternative emerges (23). If Kuhn is even partly right, falsification cannot be left to individual virtue. It must be built into the institutions of science, through support for high-risk alternative hypotheses, publication of well-designed negative results, and review practices that reward rigorous attempts at refutation as much as confirmation.

## Training Must Protect the Seam Between Doing and Thinking

Addressing these broader challenges may require changing how we train future scientists. Current training often encourages researchers to specialize in either complex experimental methods or theoretical thinking, with limited interaction between the two. One possible approach is to support both paths. The experimental path would emphasize rigorous study design and standardized methods. The theoretical path would focus on integrating concepts, building mechanistic models, and testing hypotheses. Modern science is too complex for every scientist to master both areas. However, researchers should understand the other area well enough to collaborate effectively. Neither group should be isolated nor valued above the other. This proposal is a general direction rather than a detailed plan. Its main point is that experimental and theoretical scientists must be able to work together.

Many of the figures who shaped science, Bacon and Popper among them, insisted that doing and thinking cannot be separated. The data-to-wisdom failures described above occur not within experiment or within theory, but in the translation between them. Whatever its’ institutional form, the aim is only this: to protect the seam between doing and thinking, so that neither experimental rigor nor theoretical depth is euthanized to the pressure for rapid output. These failures, viewed through the DIKW paradigm, each correspond to a broken junction: D→I failures arise when experimental tracks generate data without the statistical discipline to organize it into unambiguous information; I→K failures arise when theoretical tracks lack the empirical grounding to convert structured information into causal knowledge; and K→W failures arise when neither track has the cross-disciplinary reach to abstract universal principles from domain-specific knowledge. Structural reform of scientific training must therefore target these junctions explicitly, not as an abstract ideal but as a concrete design criterion for curricula, collaborations, and evaluation frameworks.

Modern science has become too complex for a single individual to handle. It relies on a team effort, in which distinct domains of knowledge are synthesized to arrive at wisdom. Cooperation in particle physics has yielded one of the most precisely validated theories in the history of science: quantum field theory (24, 25, 26), whose predictions (for instance, the electronʼs anomalous magnetic moment) agree with experiment to more than ten significant figures (27) — a monumental testament to the power of team science. In biology, team science is a relatively new approach that has yet to fully permeate the field. To pursue bold problems that span eleven orders of magnitude in time and length scales, as is typical in biology, scientists must learn to evolve their scientific approach.

Science remains our most powerful method for understanding the natural world, but its power is derived from its humility. We must remain mindful that our models are approximations, useful at a certain operational stage, but always subject to revision. By slowing our pace to ensure information is truly distilled into wisdom, educating the next generation of scientists, and returning to the rigorous practice of seeking to refute our own best ideas, we can preserve the integrity of the scientific endeavor for generations to come. None of this is guaranteed by human involvement alone: data and models advance science only when the people who use them exercise prudent judgment, including the humility to doubt their own conclusions as readily as they do their algorithmsʼ. The DIKW hierarchy has served as the argumentʼs organizing thread throughout: each pathology examined here is a failure to complete one of its transitions, and each remedy proposed is a way of completing it. To pursue wisdom in science is to treat that ascent not as a metaphor but as a working discipline in every experiment, and in every conversation across disciplinary lines.

## Supplementary Material

Reviewer comments
