# Supplementary material for: From data to wisdom: A new era of scientific inquiry
Source: Life Sci Alliance. 2026 Oct 19;10(1):e202603865. doi: 10.26508/lsa.202603865 (PMC13680813; doi:10.26508/lsa.202603865)
Supplement: Supplementary file 1 [file LSA-2026-03865_review_history.pdf]

From Data to Wisdom: A New Era of Scientific Inquiry

Nikolay Dokholyan
DOI: https://doi.org/10.26508/lsa.202603865

Corresponding author(s): Nikolay Dokholyan, University of Virginia

**Review Timeline:**		
	Submission Date:	2026-07-29
	Editorial Decision:	2026-09-11
	Revision Received:	2026-09-28
	Editorial Decision:	2026-10-01
	Revision Received:	2026-10-02
	Accepted:	2026-10-06

Scientific Editor: Tim Fessenden

Transaction Report:

(Note: With the exception of the correction of typographical or spelling errors that could be a source of ambiguity, letters and reports are not edited. The original formatting of letters and referee reports may not be reflected in this compilation.)

No full Peer Review Process File is available with this article, as the authors have chosen not to make the review process public in this case.

September 11, 2026

Re: Life Science Alliance manuscript #LSA-2026-03865-T

Prof. Nikolay V Dokholyan
University of Virginia
Departments of Neurology, Neuroscience, Pharmacology, Biomedical Engineering, Microbiology, Immunology, & Cancer Biology
Charlottesville, Virgina 22903

Dear Dr. Dokholyan,

Thank you for submitting your perspective entitled "From Data to Wisdom" to Life Science Alliance. We circulated your work to two expert reviewers, whose comments are appended to this letter.

We invite you to consider the remaining suggestions from both reviewers towards improving this work, although we feel that a bulleted list of key points is not needed.

A revised manuscript will be swiftly considered at the editorial level without further input from reviewers, unless you prefer otherwise. In that case kindly let us know.

While you are revising your manuscript, please also attend to the below editorial points to help expedite the publication of your manuscript. Please direct any editorial questions to the journal office.

To upload the revised version of your manuscript, please log in to your account: https://lsa.msubmit.net/cgi-bin/main.plex
You will be guided to complete the submission of your revised manuscript and to fill in all necessary information. Please get in touch in case you do not know or remember your login name.

A. THESE ITEMS ARE REQUIRED FOR REVISIONS

-- A letter addressing the reviewers' comments point by point.

-- An editable version of the final text (.DOC or .DOCX) is needed for copyediting (no PDFs).

-- Summary blurb (enter in submission system): A short text summarizing in a single sentence the study (max. 200 characters including spaces). This text is used in conjunction with the titles of papers, hence should be informative and complementary to the title. It should describe the context and significance of the work for a general readership; it should be written in the present tense and refer to the work in the third person. Author names should not be mentioned.

B. MANUSCRIPT ORGANIZATION AND FORMATTING:

Full guidelines are available on our Instructions for Authors page, https://www.life-science-alliance.org/authors

We hope that the comments below will prove constructive as your work progresses. We would be happy to discuss them further once you've had a chance to consider the points raised in this letter.

Thank you for this interesting contribution to Life Science Alliance. We are looking forward to receiving your revised manuscript.

Sincerely,

Guangshuo Ou
Monitoring Editor
Life Science Alliance

October 1, 2026

RE: Life Science Alliance Manuscript #LSA-2026-03865-TR

Prof. Nikolay V Dokholyan
University of Virginia
Departments of Neurology, Neuroscience, Pharmacology, Biomedical Engineering, Microbiology, Immunology, & Cancer Biology
Charlottesville, Virginia

Dear Dr. Dokholyan,

Thank you for submitting your revised manuscript entitled "From Data to Wisdom". We have evaluated the rebuttal letter and revised manuscript at the editorial level. With gratitude to our reviewers, we are glad to offer provisional acceptance of this work in Life Science Alliance.

Please upload the following materials to our submission system by logging in to your account: https://lsa.msubmit.net/cgi-bin/main.plex
You will be guided to complete the submission of your revised manuscript.

These items are required for acceptance.

-- An editable version of the final text (.DOC or .DOCX) is needed for copyediting (no PDFs).

-- High-resolution figure, supplementary figure and video files uploaded as individual files: See our detailed guidelines for preparing your production-ready images, https://life-science-alliance.org/authorguide

-- Summary blurb (enter in submission system): A short text summarizing in a single sentence the study (max. 200 characters including spaces). This text is used in conjunction with the titles of papers, hence should be informative and complementary to the title. It should describe the context and significance of the findings for a general readership; it should be written in the present tense and refer to the work in the third person. Author names should not be mentioned.

The license to publish form must be signed before your manuscript can be sent to production. A link to the license to publish form will be sent to the corresponding author only. Please take a moment to check your funder requirements.

Thank you for your attention to these final processing requirements. Please revise and format the manuscript and upload materials within 7 days.

Thank you for this interesting contribution, we look forward to publishing your Research Article in Life Science Alliance.

Sincerely,

Tim Fessenden
Scientific Editor
Life Science Alliance

October 6, 2026

RE: Life Science Alliance Manuscript #LSA-2026-03865-TRR

Prof. Nikolay V Dokholyan
University of Virginia
Departments of Neurology, Neuroscience, Pharmacology, Biomedical Engineering, Microbiology, Immunology, & Cancer Biology
Charlottesville, Virginia

Dear Dr. Dokholyan,

Thank you for submitting your revised manuscript entitled "From Data to Wisdom: A New Era of Scientific Inquiry". I must apologize for the time needed on our end to send this official notice of acceptance. It is a pleasure to let you know, at last, that your commentary is now accepted for publication in Life Science Alliance. Congratulations on this stimulating and valuable meditation on the research enterprise in biology, which I am sure our readers will appreciate.

The final published version of your manuscript will be deposited by us to PubMed Central upon publication.

Your manuscript will now progress through copyediting and proofing. It is journal policy that authors provide original data upon request.

Your article will publish open access upon publication under a CC-BY license.

Reviews, decision letters, and point-by-point responses associated with peer-review at Life Science Alliance will be published, alongside the manuscript. If you do want to opt out of having the reviewer reports and your point-by-point responses displayed, please let us know immediately.

IMPORTANT: If you will be unreachable at any time, please provide us with the email address of an alternate author. Failure to respond to routine queries may lead to unavoidable delays in publication.

Scheduling details will be available from our production department. You will receive proofs shortly before the publication date. Only essential corrections can be made at the proof stage so if there are any minor final changes you wish to make to the manuscript, please let the journal office know now.

DISTRIBUTION OF MATERIALS:
Authors are required to distribute freely any materials used in experiments published in Life Science Alliance. Authors are encouraged to deposit materials used in their studies to the appropriate repositories for distribution to researchers.

You can contact the journal office with any questions, contact@life-science-alliance.org

Again, congratulations on a very nice commentary. I hope you found the review process to be constructive and are pleased with how the manuscript was handled editorially.

Sincerely,

Tim Fessenden
Scientific Editor
Life Science Alliance
